# Real-world Biapenem vs. Meropenem in the treatment of severe community-acquired pneumonia in children: A propensity score matching analysis

**DOI:** 10.3389/fped.2022.1047595

**Published:** 2022-11-21

**Authors:** Xuemei Tao, Changjing Xu, Xiaoyan Zhong, Yao Mou, Jingwei Li, Xuping Yang, Yilan Huang

**Affiliations:** ^1^ Department of Pharmacy, The Affiliated Hospital of Southwest Medical University, Luzhou, China; ^2^ School of Pharmacy, Southwest Medical University, Luzhou, China

**Keywords:** Biapenem, severe community-acquired pneumonia, propensity score matching method, children, efficacy, safety

## Abstract

**Objective:**

To compare the real-world efficacy and safety of Biapenem and Meropenem for treating severe community-acquired pneumonia (SCAP) in children.

**Methods:**

We retrospectively evaluated 915 children with SCAP who were treated with Biapenem or Meropenem from August 2018 to June 2022. A 1:1 propensity score matching (PSM) analysis was used to reduce the actual baseline difference between groups.

**Results:**

416 patients participated in the analysis after PSM (Biapenem: Meropenem = 1:1). For the Biapenem group and Meropenem group, the effective rates were 90.4% and 90.9%, respectively (*p *= 1.0) and the incidence of adverse reactions were 7.7% and 7.2%, respectively (*p *= 1.0). There were no statistical differences between Biapenem and Meropenem.

**Conclusion:**

In general, the efficacy and safety of Biapenem are comparable to Meropenem in the treatment of children with SCAP.

## Introduction

Community-acquired pneumonia (CAP) is the main cause of children's hospitalization and death. The World Health Organization (WHO) estimated that the incidence of CAP in low-and middle-income countries' children in 2010 was approximately 0.22 cases per child-year, of which 11.5% of cases developed severe pneumonia (1). Children with severe pneumonia may develop a variety of pulmonary complications, such as pneumothorax, empyema, pulmonary abscess, acute respiratory distress syndrome (ARDS), and even chronic respiratory failure. Severe respiratory infection before the age of 5 has a considerable adverse effect on adult lung function and COPD (2). Therefore, once severe community-acquired pneumonia (SCAP) is diagnosed, timely and effective treatment should be carried out in children to reduce the mortality and sequelae (3). Success in the treatment of SCAP depends on timely provision of antibiotics or antivirals against potentially causative microorganisms (4). Some studies have shown that the pathogenic bacteria of SCAP mainly include viruses, bacteria, chlamydia and mycoplasma (5, 6). The American Guideline of CAP recommended that for selected critically ill patients, in addition to the core microorganisms of the CAP, empirical treatment should also be carried out for such pathogens as methicillin-resistant Staphylococcus aureus (MRSA), Pseudomonas aeruginosa and other drug-resistant gram-negative bacteria (7). The Chinese guideline for the diagnosis and treatment of CAP for children (2019 edition) suggested that Imipenem or Meropenem could be used when fatal complications or extended-spectrum β-lactamase-producing bacteria (ESBLs) were considered (8).

Biapenem is a novel type of parenteral broad-spectrum carbapenem, which has been used for the treatment of sepsis, lower respiratory tract infection, genitourinary system infection, abdominal cavity and urinary tract infection in Japan, Thailand and China for nearly 20 years (9). However, there are few studies on Biampenem for children, and it is not mentioned in the childhood infectious disease guidelines. Herein, to provide evidence for clinical medication, we first retrospectively analyzed the efficacy and safety of Biapenem and Meropenem in treating children with SCAP.

## Materials and methods

### Patients

This retrospective case-control study included children diagnosed as SCAP in the Department of Pediatrics, Affiliated Hospital of Southwestern Medical College, from August 2018 to June 2022. Patient clinical data came from hospital information system. CAP was defined according to the guidelines of the American Thoracic Society and the American Society of Infectious Diseases in 2007 (7). The diagnosis of SCAP based on guidelines for the diagnosis and treatment of CAP for children (2019 edition) (8). The inclusion criteria were (1) age range from 29 days to 18 years; (2) the patients received 10–20 mg/kg q8h Biapenem or Meropenem by intravenous drip. Patients were excluded if (1) hospital acquired pneumonia; (2) non-infectious pneumonitis, such as aspiration, uremic, hypersensitivity pneumonitis; (3) the course of treatment with Biapenem or Meropenem is too short (less than three days); (4) cases with incomplete data. This study was approved by the Southwest Medical College Hospital Ethics Committee (No. KY2022289).

### Data collection

The clinical data collected were pre-determined based on clinical experience and literature reviewand. Forms were created to collect data such as gender, age, consciousness (somnolence, coma, convulsions), complications (8), underlying diseases (8), invasive mechanical ventilation (IMV) and laboratory results sunch as procalcitonin (PCT), white blood cell count (WBC) and C-reactive protein (CRP).

### Evaluation of clinical efficacy

The efficacy evaluation criteria refer to the Guidelines for the Diagnosis and Treatment of Cough (2015 Edition) (10), and The Chinese guideline for the diagnosis and treatment of CAP for children (2019 edition) (8), comprehensively formulated as: (1) Recovery, the symptoms and signs disappeared when the child was discharged from the hospital; (2) improvement, the symptoms and signs of the child were improved when discharged, but did not completely disappear; (3) the symptoms and signs of the child did not improved when discharged, and the family gave up the treatment; (4) child death. (1) and (2) were judged to be effective.

### Safety evaluation

A physician or pharmacist classifies adverse reactions according to their causal relationship (unrelated, probably not, possibly, probably, or definitely related) to the study drug. An adverse reaction was considered causal if it is classified as either possibly, probably, or definitely related (11).

### Propensity score matching

To decrease the impact of different baseline characteristics between the Biapenem group and the Meropenem group, we adjusted for confounding factors using PSM, a reliable method for adjusting for confounders in observational studies (12). To calculated the propensity scores, we used the following variables: Age, sex, IMV, underlying disease, complication, disturbance of consciousness, three depression sign, WBC, PCT, CRP. Matches were processed by the nearest neighbor algorithm, and the caliper width is 0.1 times the Logit standard deviation (SD) of the tendency score. Match quality was checked by absolute normalized mean difference between groups after matching, and values less than 0.1 were considered to be equally distributed between groups (13).

### Statistical methods

We compared differences among baseline variables, the effective rate, incidence of adverse reactions before and after matching. Shapiro-Wilk test was used to test the normality of Continuous variables. The mean ± SD and 25–75th percentiles were used to describe continuous normal variables and non-normal variables respectively. For categorical variables, data are presented as counts or percentages. Wilcoxon's rank-sum test and Student's test were used to compare non-normal and normal continuous variables respectively. Differences in categorical variables were tested by Fisher's exact test. Two-sided differences at *p *≤ 0.05 were considered statistically significant. All analyses were performed using R software (version 4.0.3) (R Foundation for Statistical Computing, 2020).

## Results

A total of 915 cases were analyzed, including 211 Biapenem treated cases and 704 Meropenem treated cases. After PSM, 416 patients were included in the analysis (Biapenem: Meropenem = 1:1).

### Baseline data

**Figure 1:**
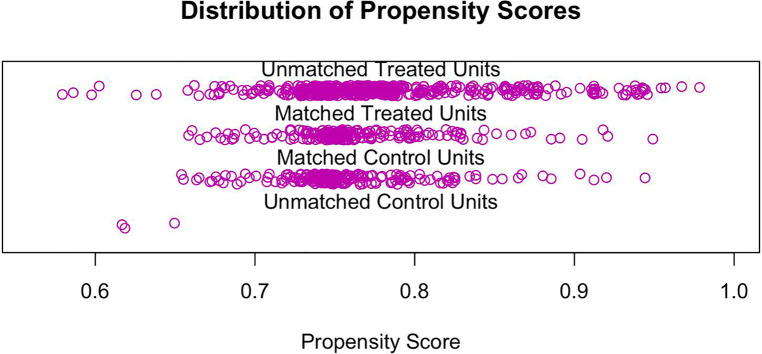
Matching jitter chart of bias scores between Beapenem group and Meropenem group.

Before PSM, age and IMV were statistically different between the Biapenem and Meropenem groups. After PSM, all baseline characteristics were balanced between two groups: Gender (proportion of males, 65.4% vs. 67.8%, *p *= 0.678), Age [20.75 (27.38) vs. 22.32 (27.86)), *p *= 0.563], Underlying disease (positive, 35.6% vs. 39.4%, *p *= 0.478), IMV (positive, 1.9% vs. 2.9%, *p *= 0.749), complication (positive, 16.3% vs. 19.7%, *p *= 0.444), disturbance of consciousness (positive, 3.4% vs. 5.3%, *p *= 0.47), WBC (positive, 59.6% vs. 62.0%, *p *= 0.688), PCT (positive, 16.3% vs. 15.9%, *p *= 1), CRP (positive, 26.4% vs. 31.2%, *p *= 0.33), three depression sign (positive, 40.4% vs. 44.2%, *p *= 0.487) (Table 1). After matching, 416 cases were included in the PSM model. All covariates were all well matched, there were no statistical difference (*p *> 0.05) (Figure 1).

**Table 1:** Comparison of baseline data between Biapenem group and Meropenem group before and after matching.

Variables, *n* (%) or mean ± S.D.	Original cohort (*n* = 915)			Matched cohort (*n* = 416)		
	Biapenem group (*n* = 211)	Meropenem group (*n* = 704)	*p*	Biapenem group (*n* = 208)	Meropenem group (*n* = 208)	*p*
Gender			1.0			0.678
Male	137 (64.9)	459 (65.2)		136 (65.4)	141 (67.8)	
Female	74 (35.1)	245 (34.8)		72 (34.6)	67 (32.2)	
Age (month)	20.60 (27.22)	26.68 (33.85)	0.017	20.75 (27.38)	22.32 (27.86)	0.563
Underlying diseases			0.585			0.478
Yes	75 (35.5)	267 (37.9)		74 (35.6)	82 (39.4)	
No	136 (64.5)	437 (62.1)		134 (64.4)	126 (60.6)	
IMV^①^			0.013			0.749
Yes	4 (1.9)	47 (6.7)		4 (1.9)	6 (2.9)	
No	207 (98.1)	657 (93.3)		204 (98.1)	202 (97.1)	
Complication			0.323			0.444
Yes	36 (17.1)	144 (20.5)		34 (16.3)	41 (19.7)	
No	175 (82.9)	560 (79.5)		174 (83.7)	167 (80.3)	
Disturbance of consciousness			0.471			0.47
Yes	10 (4.7)	45 (6.4)		7 (3.4)	11 (5.3)	
No	201 (95.3)	659 (93.6)		201 (96.6)	197 (94.7)	
WBC^②^			0.997			0.688
Abnormal	127 (60.2)	426 (60.5)		124 (59.6)	129 (62.0)	
Normal	84 (39.8)	278 (39.5)		84 (40.4)	79 (38.0)	
PCT^③^			0.334			1.0
Abnormal	36 (17.1)	99 (14.1)		34 (16.3)	33 (15.9)	
Normal	175 (82.9)	605 (85.9)		174 (83.7)	175 (84.1)	
CRP^④^			0.482			0.33
Abnormal	57 (27.0)	210 (29.8)		55 (26.4)	65 (31.2)	
Normal	154 (73.0)	494 (70.2)		153 (73.6)	143 (68.8)	
Three Depression Sign			0.69			0.487
Yes	86 (40.8)	274 (38.9)		84 (40.4)	92 (44.2)	
No	125 (59.2)	430 (61.1)		124 (59.6)	116 (55.8)	

Note: ① IMV, invasive mechanical ventilation; ② WBC <4 × 10^9^/L or >10 × 10^9^/L is abnormal; ③ PCT >2 μg/L is abnormal (14); ④ CRP >20 mg/L is abnormal.

### Clinical outcomes and adverse reactions

#### The effective rate

Before matching, the effective rate was 90% in the Biapenem group and 90.3% in the Meropenem group. After matching, the effective rate of the Biapenem group and the Meropenem group were 90.4% and 90.9%, respectively (*p *= 1.0). No statistical differences exist between groups before and after matching (Table 2).

**Table 2:** Comparison of clinical outcomes and adverse reactions between Biapenem group and Meropenem group before and after matching.

Variables, *n* (%)	Original cohort			Matched cohort		
	Biapenem (*n* = 211)	Meropenem (*n* = 704)	*p*	Biapenem (*n* = 208)	Meropenem (*n* = 208)	*p*
Effective	190 (90.00)	636 (90.3)	1.0	188 (90.4)	189 (90.9)	1.0
ADR	16 (7.6)	49 (7.0)	0.876	16 (7.7)	15 (7.2)	1.0

#### The incidence of adverse reactions

The most common adverse reactions in the experimental and control groups were diarrhea and rash. Before matching, the incidence of adverse reactions of Biapenem and Meropenem were 7.6% and 7.0% respectively. After matching, the incidence of adverse reactions for the Biapenem and Meropenem groups were 7.7%, 7.2%, respectively (*p *= 1.0). No statistical differences exist between the two groups before and after matching (Table 2).

## Discussion

In this retrospective studies, Biapenem was comparable to Meropenem in terms of efficacy and safety in children with SCAP. Additionally, Biapenem was generally well tolerated, and the most common adverse reactions were rash and diarrhea.

The results showed that the proportion of children under 1 year old was 58.41% (*n* = 243), indicating that SCAP in children mainly occurs in infancy, which is similar to the results of Long Yuwen et al. (15). In another study, SCAP was also more common in infants aged 2–12 months (16). However, Jain S et al. found the age of the children with CAP was 2(1,6) years (median, Q1,Q3) (17). The discrepancy may be due to geographic or ethnic differences, and part of the population in Jain S et al.' study had non-severe pneumonia.

Some studies (18–20) have shown that the most common pathogens in CAP patients are Streptococcus pneumoniae and respiratory viruses. Other common pathogens include Staphylococcus aureus, Moraxella catarrhalis and Haemophilus influenzae, as well as atypical microorganisms such as Chlamydophila pneumonia, Mycoplasma pneumoniae. In addition, about 5%–30% of CAP is caused by gram-negative bacteria (21). Klebsiella pneumoniae (KP) and Escherichia coli (E. coli) are not common pathogens of CAP, while they can cause SCAP (8, 22), more common in infants, or those with underlying diseases such as chronic inhalation, congenital heart disease, airway malformation, immunocompromised, and severe viral infection (8). Important issues in treatment include timely initiation of appropriate antibiotics or antivirals. Empirical antibiotic therapy should analyze possible pathogens and formulate a reasonable treatment plan based on age, epidemiology, clinical and imaging manifestations, disease severity, underlying diseases, and laboratory test results (8, 23). Current literature and clinical practice showed that carbapenems or cefoperazone/sulbactam can be used when the infections may be caused by E.coli or KP for patients with SCAP. In our study, 416 patients were SCAP, and symptoms, signs, laboratory tests, radiographic images supported a possible bacterial infection; 37.5% children had at least one underlying disease, the most common of which was congenital heart disease; the proportion of children under 1 year old was 58.41%. Therefore, pneumonia caused by gram-negative bacteria could not be excluded, and 416 children had indications for carbapenem.

The carbapenem antibiotics are regarded as the most potent antimicrobial agents with broad antibacterial activities. Representative drugs include Imipenem, Meropenem, Ertapenem, Biapenem, Panipenem etc. Carbapenems were the mainstay of treatment for multidrug-resistant (MDR) gram-negative bacteria caused serious infections, especially those caused by expressing ESBL or Ampc-type enzymes (24–26). Compared with other carbapenems, Biapenem cannot be excreted by the efflux pump of Pseudomonas aeruginosa or Baumannii, and is less likely to be resistant to resistance (27). For the hydrolysis of renal dihydropeptidase-I (DHP-I), Biapenem is more stable than imipenem and panipenem, and does not to require concomitant use of DHP-I inhibitors (28). Its triazole cationic structure also endows it with good adventitial permeability, enabling it to penetrate various body fluids (e.g., sputum, ascites, pleural effusion) and tissues (e.g., lung tissue); at the same time, Biapenem has a lower convulsive potential and can decrease the risk of seizures (29). In some vitro studies (30–33), Biapenem against gram-positive bacteria was almost the same as that of Imipenem, and the inhibitory effect on gram-negative bacteria (such as Enterobacter cloacae, Lemonella freundii and Salmonella marcescens) is better, including Enterobacter ESBL and Pseudomonas aeruginosa. The *in vitro* inhibitory effect of Biapenem against drug-resistant Pseudomonas aeruginosa was superior to Meropenem (34).

Our study results were similar to the following studies on the primary end-point. A randomized, multicenter, parallel controlled clinical trial was conducted in 9 centers in China to compare the efficacy and safety of Biapenem and Meropenem in the treatment of urinary tract infections and bacterial lower respiratory tract in adults. The overall effective rates of Biapenem and Meropenem were 94.70% and 93.94% respectively, with no significant difference. In addition, the incidence of adverse reactions caused by drugs was similar for Biapenem (11.76%) and Meropenem (15.44%) (35). Ma Xiaohua's systematic review also showed that, comparing Biapenem with Meropenem or Imipenem/cilastatin in adult, the total efficiency [RR = 1.04, 95% CI (0.98, 1.10), *p *= 0.19] and the incidence of adverse reactions [RR = 0.83, 95% CI (0.60, 1.15), *p *= 0.26] had no significant differences (36).

Our study showed that the most common adverse reactions of Biapenem or Meropenem were diarrhea and rash; the incidence of adverse reactions in the Biapenem group and Meropenem group was 7.7% and 7.2%, respectively, which were higher than previous reports (37, 38). An analysis of safety data in 2,323 patients treated with Biapenem showed rash and diarrhea in 1.0% and 0.5% of patients, respectively (37). Nausea, vomiting, and rash had been reported in less than 3% for Meropenem (38). This discrepancy may be due to different study populations. Gastrointestinal adverse reactions of antibiotics are especially prone to occur in children, the elderly and severe patients. In this study, both Meropenem and Biapenem group had good central nervous system tolerability, which was consistent with previous reports.

Although only a retrospective study, we used PSM to balance the baseline data of the two groups of patients to minimize the effect of baseline differences on the results. However, there were limitations in this study. Duration of antibiotics, bacterial eradication rates and length of hospital stay were not discussed as secondary endpoints. Due to the retrospective setting of this study, some clinical and laboratory data such as transaminases were not available from medical record review; adverse reactions were also limited to clinical symptoms. Finally, this study is a retrospective single-center study with a small sample size, and further multi-center validation studies are needed.

In conclusion, the study showed that Biapenem was noninferior to Meropenem with good tolerance. Biapenem may be an alternative treatment option for children with SCAP.

## Data Availability

The original contributions presented in the study are included in the article/Supplementary Material, further inquiries can be directed to the corresponding author/s.
